# Early Body-Composition Trajectory During Tirzepatide Treatment for Obesity: A Real-World Bioimpedance Cohort Study

**DOI:** 10.3390/jcm15218377

**Published:** 2026-10-28

**Authors:** Enes Zafer, Deniz Çekiç, Ahmed Bilal Genç, Selçuk Yaylacı

**Affiliations:** 1 Department of Internal Medicine, Sakarya University Training and Research Hospital, 54100 Sakarya, Turkey; ezafer@sakarya.edu.tr (E.Z.); decekic@gmail.com (D.Ç.); 2 Department of Internal Medicine, Faculty of Medicine, Sakarya University, 54100 Sakarya, Turkey; ahmedbgenc@gmail.com

**Keywords:** tirzepatide, obesity, body composition, bioelectrical impedance, fat-free mass, fat-free mass index, real-world evidence

## Abstract

**Background/Objectives:** Whether the loss of fat-free mass (FFM) during tirzepatide treatment exceeds the proportion expected in negative energy balance is debated, and the earliest weeks of treatment are the least characterized. We described the body-composition trajectory over the first 12 weeks of treatment in routine care. **Methods:** In this retrospective cohort of adults with obesity starting tirzepatide monotherapy, body weight, fat mass, FFM, total body water (TBW), and waist circumference were measured by single-frequency, foot-to-foot bioelectrical impedance analysis (BIA) at baseline and at approximately 4, 8, and 12 weeks, and were analyzed with linear mixed-effects models; follow-up values are available-data, on-treatment estimates. **Results:** Of 476 patients (mean age 40 years, 66% female, mean body-mass index 38.7 kg/m^2^), 268 had follow-up data and 111 were measured at 12 weeks. Estimated marginal body weight fell by 11.22 kg (10.4%), fat mass by 7.90 kg (16.8%), and BIA-derived FFM by 3.35 kg (5.4%) at 12 weeks (all *p* < 0.001); assuming 5-kg-less loss in patients without a 12-week measurement, the mean loss remained 7.4 kg. Of patients measured at 12 weeks, 89.2% had lost at least 5% and 57.7% at least 10% of body weight. In patients with complete data at baseline and 12 weeks (n = 85), fat mass accounted for 68.3% (95% CI 56.1 to 80.8) of the weight lost. **Conclusions:** Early weight loss during tirzepatide treatment was predominantly fat mass, with a BIA-derived FFM fraction numerically close to the commonly cited one-fourth. Because device-FFM equaled TBW divided by a fixed coefficient of 0.731 at every visit, it cannot separate water from lean tissue and is best read as an upper bound on lean tissue loss rather than a measure of muscle.

## 1. Introduction

Obesity is a chronic, relapsing disease and a major driver of cardiometabolic morbidity, and sustained weight loss beyond 10% improves or prevents type-2 diabetes, hypertension, dyslipidemia, metabolic dysfunction associated steatotic liver disease, and obstructive sleep apnea [1]. Recent work has moved the field from a body-mass index threshold towards a definition anchored in organ dysfunction and functional limitation, which places tissue-level outcomes rather than weight alone at the center of treatment assessment [2]. Contemporary guidelines recommend pharmacotherapy alongside nutrition and physical activity [1].

Incretin-based agents have transformed obesity pharmacotherapy. The glucagon-like peptide-1 (GLP-1) receptor agonist semaglutide and the dual glucose-dependent insulinotropic polypeptide (GIP) and GLP-1 receptor agonist tirzepatide produced mean total weight reductions of roughly 15% and 21% in phase three trials [3,4]. Tirzepatide was approved for chronic weight management in November 2023 [5], produced greater weight loss than semaglutide in a head-to-head trial [6], reduced progression to type-2 diabetes over three years [7], and required continued treatment to maintain the reduction achieved [8]. As these agents are adopted at scale, attention has shifted from the magnitude of weight loss to its composition, and specifically to the balance between fat mass and lean tissue.

The reference expectation is that approximately one-fourth of the weight lost during negative energy balance is fat-free mass, a rule whose own authors emphasized its dependence on the starting adiposity and the rate of loss [9]. In the dual-energy X-ray absorptiometry (DXA) substudy of SURMOUNT-1, tirzepatide reduced fat mass by 33.9% against 10.9% for lean mass, and the fat and lean shares of the loss (about 75% and 25%) were similar to placebo [10]. That reassuring picture is not uncontested. A systematic review of 35 randomized trials reported a median muscle-related fraction of weight loss of 28.3% (interquartile range 15.9 to 39.9) with incretin therapy, with about two-thirds of estimates exceeding the one-quarter benchmark and a higher fraction derived from bioimpedance or DXA (median about 29%) than from computed tomography or magnetic resonance imaging (median about 25.3%); comparison groups on lifestyle intervention exceeded the benchmark less often (about 38%), and, importantly for the present study, no included study reported an objective measure of physical function [11]. A network meta-analysis of 22 trials placed lean mass loss at 0.86 kg against 2.95 kg of fat, close to one-quarter of total loss [12], whereas narrative syntheses report fat-free mass fractions spanning 20–40% [13] and 15–45% [14] across agents and methods. Whether these losses are maladaptive or an expected adaptation to a smaller body remains unsettled [15,16].

Two features of the existing evidence limit its translation to clinical practice. First, most body-composition data come from trial populations assessed at 6 months or later, so the early kinetics (the period when energy deficit and fluid shift are largest) are poorly described. Second, real-world series are small and methodologically heterogeneous: a 106-patient DXA cohort on semaglutide reported an early fat-free mass decline of 3 kg that then stabilized, with improved grip strength [17]; a 52-patient bioelectrical impedance analysis (BIA) cohort on tirzepatide reported preserved fat-free mass and unchanged muscle function [18]; and a 269-patient BIA series reported muscle preservation with concurrent lifestyle support [19]. Larger real-world cohorts have characterized titration, persistence and adverse events rather than composition [20,21,22], and a foundational study of GLP-1 effectiveness in routine care used person-level mixed models that adjusted for baseline predictors of missing follow-up weight [23], the approach adopted here.

We therefore examined the early body-composition trajectory during tirzepatide treatment in a real-world obesity cohort, using serial single-frequency, foot-to-foot bioimpedance at baseline and at 4, 8, and 12 weeks. Secondary objectives were to describe accompanying metabolic changes, to test whether baseline hepatic steatosis modified the response, and to quantify explicitly how much of the observed fat-free mass signal reflects the water-based construction of the measurement.

## 2. Materials and Methods

### 2.1. Study Design and Setting

This was a retrospective cohort study of adults (aged 18 years or older) with obesity who received routine care at the obesity outpatient clinic of the Department of Internal Medicine, Sakarya University Training and Research Hospital, Sakarya, Turkey, and started tirzepatide in routine care. The clinical records included in the study covered the period from 1 September 2025 to 31 March 2026. Reporting follows the Strengthening the Reporting of Observational Studies in Epidemiology (STROBE) recommendations [24] and the RECORD extension for routinely collected health data [25].

The primary outcomes were the changes in body weight, fat mass, and fat-free mass over the first 12 weeks. Secondary outcomes were the changes in waist circumference, total body water, body-fat percentage and derived indices, the proportion of patients reaching categorical weight-loss thresholds, the changes in fasting laboratory parameters, and effect modification of the trajectory by baseline hepatic steatosis and by obesity class. Tirzepatide was started at 2.5 mg once weekly and titrated by the treating physician according to clinical response and gastrointestinal tolerability. Dose was not an eligibility criterion, and the dose received at each visit was not available in the study dataset.

### 2.2. Participants

**Figure 1:**
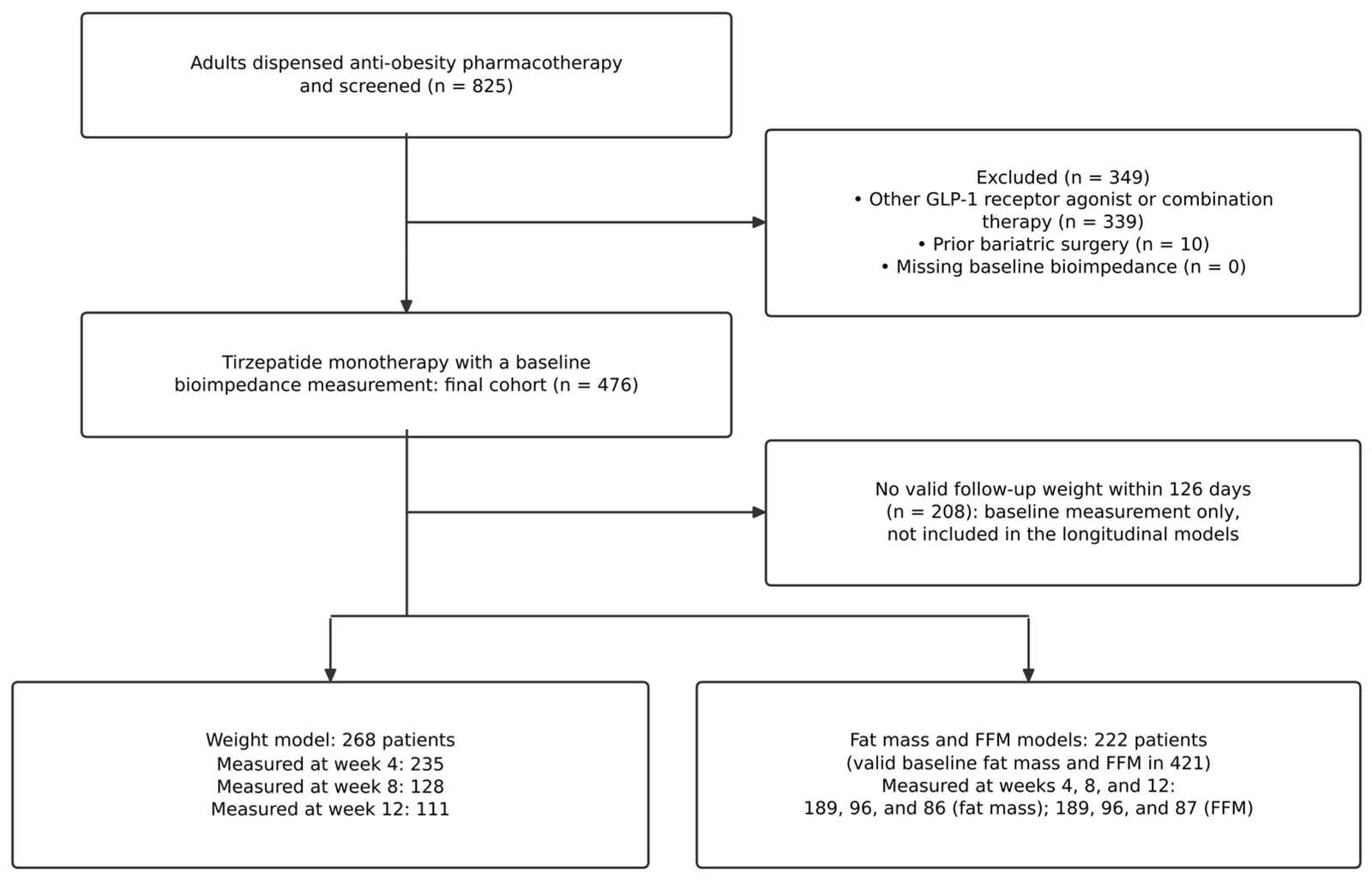
Study-cohort flow diagram. Follow-up counts are the numbers of patients with a valid measurement in each visit window.

The records of 825 adults dispensed anti-obesity pharmacotherapy were screened (Figure 1). For the estimates to reflect tirzepatide alone, 339 patients receiving another GLP-1 receptor agonist (semaglutide, liraglutide, lixisenatide) or combination anti-obesity pharmacotherapy were excluded, and 10 patients with prior bariatric surgery were excluded because previous surgery alters body composition and its bioimpedance estimation [26]. Standard bioimpedance contraindications, including pregnancy and an implanted cardiac pacemaker or defibrillator, precluded measurement and so did not enter the screened pool; advanced renal or hepatic failure and regular diuretic or corticosteroid use, which can affect fluid balance, were also eligibility exclusions. Every remaining patient on tirzepatide monotherapy had a baseline bioimpedance measurement, so none were excluded for a missing baseline. The final cohort comprised 476 patients. Inclusion criteria were age 18 years or older, obesity treated with tirzepatide monotherapy, and a baseline bioimpedance measurement; attendance at follow-up visits and baseline obesity class were not eligibility criteria. Of the 476 patients, 268 had at least one valid follow-up weight within 126 days of baseline and entered the longitudinal models, whereas 208 had a baseline measurement only (15 of them were measured again only after this window). Of the 421 patients with valid baseline fat mass and fat-free mass values, 222 had at least one valid follow-up value (Figure 1; Supplementary Table S2). The secondary analyses used the patients with the relevant data: 380 with baseline ultrasonography for the steatosis analysis and 57 to 61 with paired baseline and follow-up laboratory values.

### 2.3. Ethics

The study was approved by the Health Sciences Scientific Research Ethics Committee, Faculty of Medicine, Sakarya University (decision no. E-43012747-050.04-567458, dated 25 February 2026). The retrospective review of medical records and the extraction of data commenced only after ethics approval had been obtained; tirzepatide treatment and all measurements were part of routine clinical care and were not performed for the purposes of the study. Individual informed consent was waived owing to the retrospective design and the exclusive use of de-identified, routinely collected clinical data.

### 2.4. Measurements

Body composition was assessed with a single-frequency, four-electrode, foot-to-foot bioelectrical impedance analyzer (Tanita TBF-300, Tanita Corporation, Tokyo, Japan; 50 kHz) under a standard clinical protocol (fasting state, bladder emptied, manufacturer-specified posture) [27]. This device passes a low alternating current between the feet and derives whole-body composition from lower-body impedance using a proprietary regression equation. Recorded parameters were body weight, body-mass index, fat mass, fat-free mass, body-fat percentage, total body water, and waist circumference. Fat-free mass, lean soft tissue, and skeletal muscle mass are distinct compartments. The device reports only fat-free mass, which it estimates from total body water using a fixed hydration coefficient; FFM in this report therefore refers to this bioimpedance-derived estimate and not to lean soft tissue or skeletal muscle, a dependence we quantify in the Results and address in the Discussion. For each participant, measurements were assigned to nominal visits at baseline and at approximately 4, 8, and 12 weeks by selecting, within predefined day windows, the measurement closest to each nominal time point. Height was recorded at baseline. Waist circumference was measured with a non-elastic tape at the midpoint between the lowest rib and the iliac crest at the end of a normal expiration. Fasting venous samples were analyzed in the hospital laboratory at baseline and at follow-up for glucose, insulin, glycated hemoglobin (HbA1c), total, low-density, and high-density lipoprotein cholesterol, triglycerides, alanine aminotransferase (ALT), aspartate aminotransferase (AST), gamma-glutamyl transferase (GGT), uric acid, creatinine, thyroid-stimulating hormone (TSH), and C-reactive protein (CRP). The homeostatic model assessment of insulin resistance (HOMA-IR) was calculated as fasting glucose (mg/dL) × fasting insulin (mIU/L)/405 [28], and the estimated glomerular filtration rate (eGFR) was reported by the laboratory using the Chronic Kidney Disease Epidemiology Collaboration (CKD-EPI) 2009 equation [29]. Hepatic steatosis was graded on abdominal ultrasound as 0 (absent), 1 (mild), 2 (moderate), or 3 (severe) using standard visual criteria based on liver echogenicity relative to the renal cortex and the visibility of the intrahepatic vessel walls and the diaphragm [30]; all examinations were performed by a single radiologist as part of routine care, and intra-observer reproducibility was not assessed.

### 2.5. Derived Measures

To place absolute changes on a height-normalized scale, we computed the fat-free mass index (fat-free mass divided by height squared) and the fat-mass index (fat mass divided by height squared), and we report FFMI against published low-muscle screening thresholds [31]. We also report the fat mass to fat-free mass ratio and the ratio of total body water to fat-free mass. The fat-free fraction of loss was computed in two ways, cumulative (from baseline to each visit) and interval (between consecutive visits), because the two behave differently when a component changes non-monotonically.

### 2.6. Statistical Analysis

Continuous variables were summarized as mean plus or minus standard deviation and categorical variables as number (percentage). Body-composition trajectories were analyzed with linear mixed-effects models that included a random intercept for each participant and time (nominal visit) as a categorical fixed effect, estimated by maximum likelihood; the overall effect of time was assessed by likelihood-ratio test and estimated marginal means with 95% confidence intervals are reported at each visit. This specification uses all available measurements under a missing-at-random (MAR) assumption. The follow-up estimates are therefore available-data, on-treatment estimates for patients who returned for measurement, not the trajectory of the complete cohort. Physiologically implausible values were excluded before analysis according to prespecified plausibility ranges. Because the fat mass, fat-free mass, and body-fat percentage models were each fitted on the subset of visits with a valid value for that variable, the contributing samples differ slightly across parameters (the number contributing at each visit is reported in the footnote to the body-composition results), and the fat-mass to weight ratio implied by the fat-mass model runs approximately one percentage point above the separately modeled body-fat percentage. As only 111 of 476 patients had a weight measurement in the 12-week window, we examined whether attrition was associated with baseline characteristics and, following the approach of White and colleagues [23], refitted the weight model adjusting for baseline age, sex, and body-mass index as a sensitivity analysis. We compared baseline characteristics between patients who did and did not enter the longitudinal models and between those with and without a 12-week measurement. Because such comparisons cannot establish that data are missing at random, we also performed a delta-adjusted pattern-mixture (tipping-point) analysis [32,33]: for patients without a 12-week weight, the value predicted by the mixed model under MAR was shifted towards less weight loss by 1 to 8 kg, and the mean 12-week change was recomputed in the modeled population and in the full cohort (using the fixed-effect prediction for patients without follow-up), with 95% confidence intervals from 200 bootstrap resamples of patients. The proportion of patients reaching 5%, 10%, and 15% weight loss at each visit was computed as a complete-case sensitivity analysis, and the rate of weight loss was derived from the modeled means. Because the weight, fat mass and fat-free mass models were fitted in non-identical samples, the fat and fat-free fractions of weight loss were also estimated in a matched subset of patients with all three variables at both time points of each comparison, restricted to records in which device weight equaled fat mass plus fat-free mass within 0.5 kg (847 of 856 records), as the ratio of summed changes with 95% confidence intervals from 4000 bootstrap resamples; the individual-level fat-free fraction among patients who lost weight is reported as median (interquartile range). Paired changes in laboratory values from baseline to 12 weeks were tested with paired *t*-tests; the distribution of paired differences was examined with the Shapiro–Wilk test and, because several departed from normality, the Wilcoxon signed-rank test was also applied. Effect modification by baseline hepatic steatosis (grade 1 or higher versus grade 0) was assessed by adding a time-by-steatosis interaction and testing it by likelihood-ratio test; because a non-significant interaction is not evidence of equivalence, we also computed the smallest between-group difference the sample could detect at 80% power and report the between-group difference in 12-week change with its 95% confidence interval. Effect modification by baseline obesity class (body-mass index below 35, 35 to 39.9, and 40 kg/m^2^ or higher) was examined in the same way for body weight and for percentage weight change. A two-sided *p* value below 0.05 was considered significant; secondary and interaction tests were not corrected for multiplicity and are interpreted as exploratory. Analyses were performed in Python (version 3.12; Python Software Foundation, Wilmington, DE, USA) using the pandas, SciPy, and statsmodels libraries.

## 3. Results

### 3.1. Cohort

A total of 476 patients treated with tirzepatide monotherapy were included; baseline characteristics are shown in Table 1. The cohort had a mean age of 40 years, was 66% female, and had a mean body-mass index of 38.7 kg/m^2^. Hepatic steatosis was present on ultrasound in 73% of patients with imaging, and metabolic comorbidities such as diabetes (11%) and hypertension (3%) were infrequently recorded. Baseline body-mass index ranged from 29.2 to 68.5 kg/m^2^; 126, 188, and 162 patients had a body-mass index below 35, from 35 to 39.9, and of 40 kg/m^2^ or higher. Comorbidities were those recorded as diagnoses in the clinical record and are likely to be under-ascertained; for example, 85% of patients with available measurements had a HOMA-IR of 2.5 or higher, whereas insulin resistance was recorded as a diagnosis in 9%.

### 3.2. Body-Composition Trajectory

**Figure 2:**
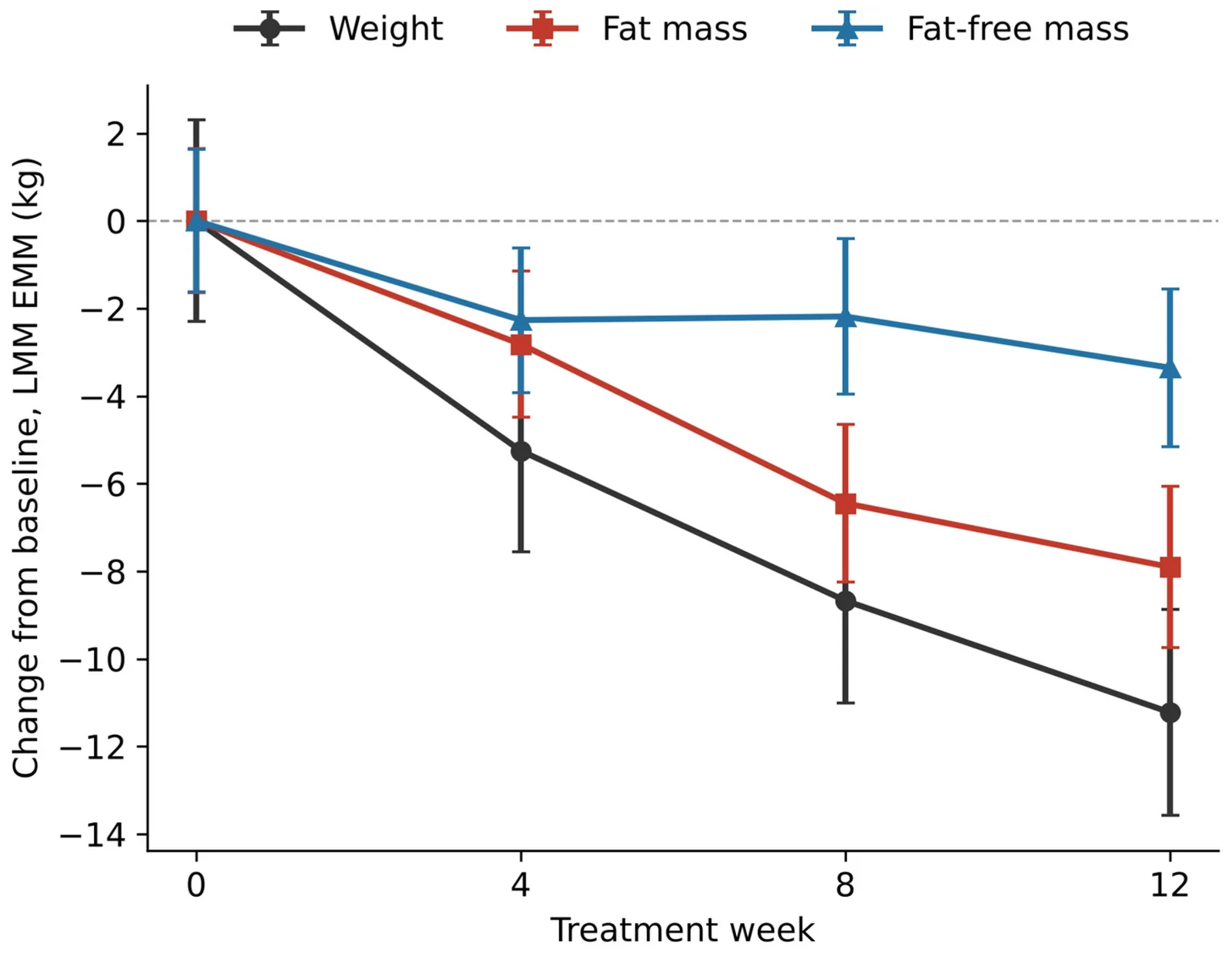
Mean change from baseline in body weight, fat mass, and fat-free mass over 12 weeks of tirzepatide treatment (estimated marginal means from linear mixed-effects models; error bars are 95% confidence intervals). Weight and fat mass decline steeply while fat-free mass changes comparatively little. The dashed horizontal line marks no change from baseline.

Body composition changed substantially over the first 12 weeks (Table 2, Figure 2). Estimated marginal body weight fell from 108.2 to 97.0 kg, a reduction of 11.22 kg (10.4%). Fat mass fell by 7.90 kg (16.8%), body-fat percentage by 3.3 points, waist circumference by 9.39 cm, BIA-derived fat-free mass by 3.35 kg (5.4%), and total body water by 2.46 kg (all *p* < 0.001). The number of patients contributing to each model ranged from 167 to 268 (contributing at baseline, week 4, week 8, and week 12: 268, 235, 128, and 111 for weight and 222, 189, 96, and 86 for fat mass). These are available-data, on-treatment estimates for the patients who returned for measurement (Figure 1, Supplementary Table S2). Compared with the 208 patients with a baseline measurement only, the 268 patients in the weight model had a slightly higher baseline weight (108.2 versus 104.0 kg), body-mass index (39.2 versus 38.0 kg/m^2^), and body fat (42.7% versus 41.1%), with similar age, sex distribution, comorbidities, and steatosis prevalence (Supplementary Table S3).

### 3.3. Categorical Weight Loss and Rate of Change

Among the 111 patients with a measurement in the 12-week window, 89.2% had lost at least 5%, 57.7% at least 10%, and 10.8% at least 15% of baseline body weight (median 10.7%); the corresponding proportions were 44.7%, 3.4%, and 0.4% at week 4 (n = 235) and 83.6%, 26.6%, and 4.7% at week 8 (n = 128) (Table 3). The rate of weight loss decelerated over the period, from 1.31 kg per week (1.21% per week) between baseline and week 4 to 0.86 kg per week between weeks 4 and 8, and 0.64 kg per week between weeks 8 and 12, a deceleration consistent with the physiology of the weight-loss plateau under GLP-1 receptor agonism [34]. Absolute 12-week weight loss increased with baseline obesity class (9.13, 10.77, and 13.03 kg for a body-mass index below 35, 35 to 39.9, and 40 kg/m^2^ or higher; interaction *p* = 0.004), whereas percentage weight loss was similar across classes (10.3%, 10.3%, and 10.2%; *p* = 0.565) (Supplementary Table S6).

### 3.4. Composition of Loss and Its Time Course

**Figure 3:**
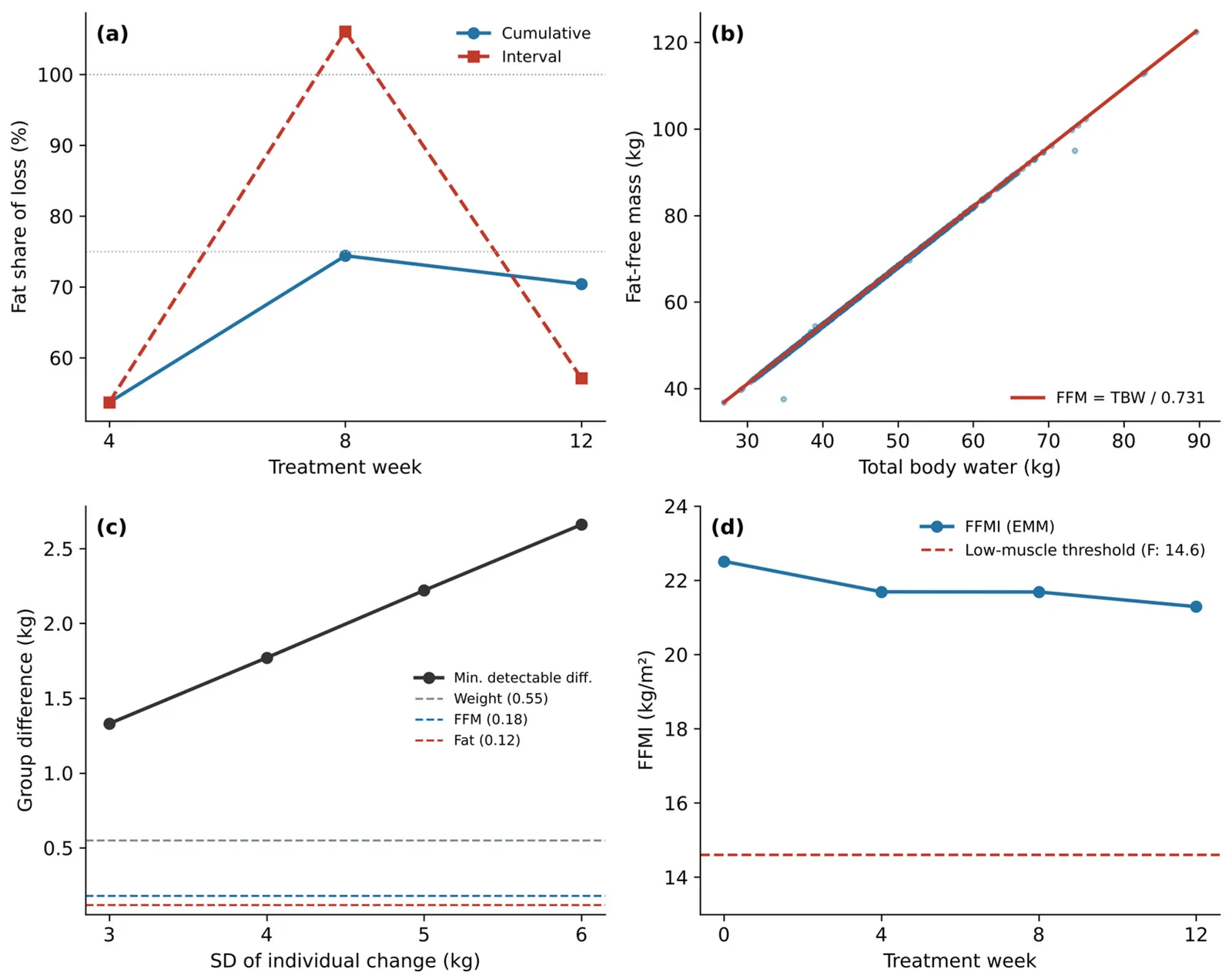
Measurement audit. (**a**) Fat share of loss derived from the marginal means, cumulative and interval; the interval share above 100% between weeks 4 and 8 reflects the modeling structure (Section 3.4). (**b**) Fat-free mass is fixed by total body water divided by 0.731 in every observation. (**c**) Smallest between-group difference in 12-week change detectable at 80% power under a range of assumptions, shown to indicate the precision of the interaction test; it is not evidence that no clinically relevant difference exists, and 95% confidence intervals for the differences are given in Section 3.8. (**d**) FFMI against a low-muscle screening threshold; the cohort remains well above it. In panel (**b**) each point is an individual observation and the red line is an identity rather than a fit; the few points departing from it reflect measurement or data-entry variation. In panel (**c**), the three dashed colored lines are the observed between-group differences and the black line is the smallest detectable difference. In panel (**a**) the dotted horizontal lines mark fat shares of 75% and 100%, and in panel (**d**) the dashed line marks the low-muscle screening threshold.

In the matched subset with weight, fat mass, and FFM at baseline and 12 weeks (n = 85), fat mass accounted for 68.3% (95% CI 56.1 to 80.8) and BIA-derived FFM for 31.7% (95% CI 19.5 to 44.1) of the weight lost, and the individual-level median FFM fraction was 28.8% (interquartile range 10.8 to 51.5); the ratio of the modeled marginal changes gave a similar split (70.4% and 29.6%) (Table 4, Supplementary Table S4). Because the device partitions weight into fat mass and FFM, the two components sum to the weight change by construction. By interval, fat accounted for 54.9% (95% CI 41.9 to 66.9) of the loss between baseline and week 4 (n = 187), 93.2% (61.1 to 131.9) between weeks 4 and 8 (n = 83), and 69.1% (17.8 to 117.0) between weeks 8 and 12 (n = 62). The first 4 weeks therefore had the least fat-selective profile, and the later interval estimates were imprecise. The interval share above 100% obtained from the marginal means between weeks 4 and 8 (Table 4, Figure 3a) reflects a small rise in the modeled FFM estimate over that interval and the use of separately fitted models, and it was not present in the matched analysis.

### 3.5. Height-Normalized Indices

Height-normalized indices showed the cohort moving towards a leaner phenotype: FFMI fell 1.22 kg/m^2^, from 22.51 to 21.29, remaining roughly 6.7 units above the thresholds used to screen for low muscle mass [31], while the fat mass to fat-free mass ratio fell from 0.769 to 0.677 (Table 4, Figure 3d).

### 3.6. Metabolic Changes

Metabolic parameters improved in the subset with paired follow-up laboratory values (Table 5). Glycated hemoglobin (5.8 to 5.3%), gamma-glutamyl transferase (31.4 to 23.9 U/L), triglycerides (178.1 to 126.6 mg/dL), total cholesterol (206.0 to 189.3 mg/dL), and uric acid (6.2 to 5.6 mg/dL) all decreased significantly by both tests, whereas aminotransferases showed smaller changes (alanine aminotransferase decreased by the Wilcoxon signed-rank test, *p* = 0.015, but not by the paired *t*-test, *p* = 0.073) and low-density and high-density lipoprotein cholesterol, creatinine, and estimated glomerular filtration rate were essentially unchanged.

### 3.7. Missing Data

Only 111 of 476 patients (23.3%) had a weight measurement in the 12-week window. Patients with and without a 12-week measurement did not differ significantly in age, sex, body-mass index, baseline weight, body fat, comorbidities, or steatosis prevalence (all *p* > 0.2; Supplementary Table S3), and adjusting the weight model for baseline age, sex, and body-mass index left the 12-week estimate essentially unchanged (adjusted change 11.21 kg versus unadjusted 11.22 kg). These findings are compatible with, but cannot establish, the missing-at-random assumption. In the delta-adjusted sensitivity analysis (Supplementary Table S5), assuming that every patient without a 12-week measurement lost 1, 3, or 5 kg less than predicted reduced the mean 12-week weight loss in the full cohort from 11.22 kg to 10.46, 8.92, and 7.39 kg, respectively. The mean loss fell below 5% of baseline weight only when a shortfall of about 8 kg was assumed, which would mean that these patients lost only about 3 kg over 12 weeks.

### 3.8. Steatosis Effect Modification

**Figure 4:**
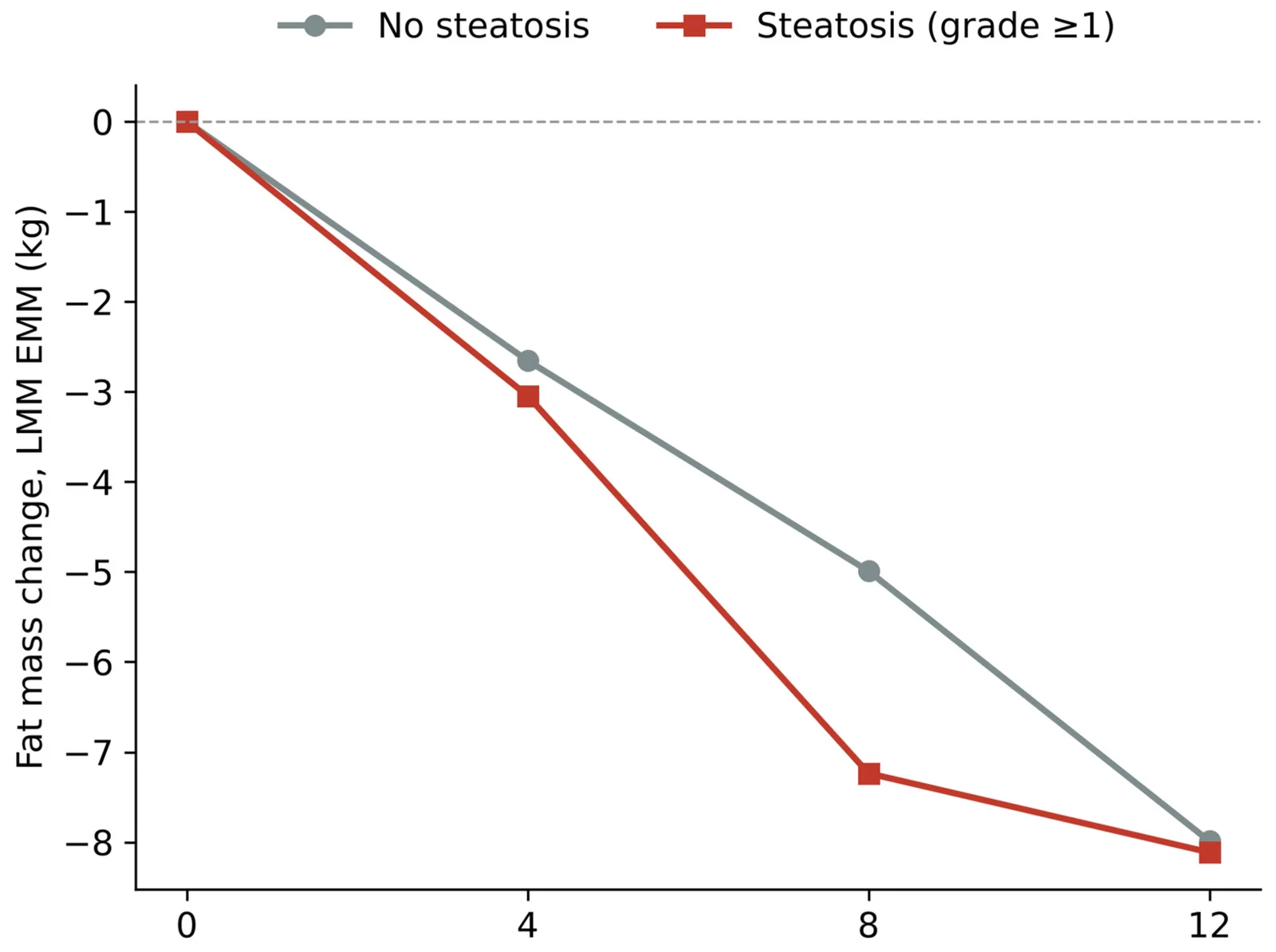
Fat-mass change over 12 weeks in patients with versus without baseline hepatic steatosis (estimated marginal means). The trajectories are closely similar and the time-by-steatosis interaction was not significant. The dashed horizontal line marks no change from baseline.

No effect modification of the body-composition trajectory by baseline hepatic steatosis was detected (Table 6, Figure 4). The 12-week changes in fat mass, fat-free mass, body weight, and body-fat percentage were closely similar between patients with and without baseline steatosis, and no interaction with time reached significance (interaction *p* of 0.32, 0.26, 0.67, and 0.41, respectively). The between-group differences in 12-week change (steatosis present minus absent) were −0.12 kg (95% CI −2.61 to 2.36) for fat mass, 0.17 kg (−2.17 to 2.52) for FFM, −0.55 kg (−1.94 to 0.85) for body weight, and 0.68 percentage points (−1.35 to 2.72) for body fat, so differences of up to about 2.5 kg in fat-mass change cannot be excluded.

## 4. Discussion

In this real-world cohort, weight loss during the first 12 weeks of tirzepatide treatment was rapid, substantial, and predominantly fat mass. In the matched analysis about 68% of the weight lost was fat and about 32% was BIA-derived FFM, a proportion close to the widely cited one-fourth rule for fat-free mass loss during negative energy balance [9] and numerically similar to the roughly 75% fat and 25% lean split reported in the DXA substudy of SURMOUNT-1 [10]; because BIA-derived FFM, DXA lean mass, and skeletal muscle are not equivalent compartments, these comparisons are contextual rather than physiological equivalences. Categorical outcomes placed the patients who remained on treatment within the range of the obesity-dose real-world literature, with 89% reaching 5% and 58% reaching 10% weight loss by 12 weeks; Supplementary Table S1 places the cohort alongside published body-composition studies of incretin therapy, as a descriptive comparison given the differences in method, agent, dose, and follow-up.

The most consequential finding of this analysis is methodological rather than clinical. The device reports fat-free mass as total body water divided by a fixed hydration coefficient of 0.731, and the reported values are reproduced exactly by that conversion at every visit. Two consequences follow. First, the 3.35 kg fall in fat-free mass and the 2.46 kg fall in total body water are two expressions of a single measurement; reporting them as corroborating findings overstates the evidence, and the approximately 30% FFM fraction is, in this data set, a restatement of water loss. Second, the constant hydration assumption is precisely the one that is least defensible in this setting: the hydration of fat-free mass varies systematically with adiposity and age [36], and rapid weight loss drives glycogen-bound and sodium-bound fluid shifts that lower it transiently, and a single-frequency 50-kHz measurement cannot resolve the intracellular from the extracellular compartment in which these shifts occur. Validation studies bear this out. Bioimpedance tracks change in fat mass well against DXA and magnetic resonance imaging but tracks change in muscle poorly (correlations of 0.24 to 0.61 for change against 0.91 to 0.99 for fat) [37], although leg-to-leg bioimpedance specifically tracks fat change during weight loss and regain with only small bias [38]; agreement against doubly labeled water after bariatric surgery is weaker for fat-free mass and water than for fat mass [39]; and impedance methods can markedly misestimate fat-free mass in the presence of weight loss and inflammation [40]; bioimpedance estimates of fat-free mass and total body water are considered valid only in the absence of significant fluid or electrolyte disturbance and with population-specific equations [41]. Agreement against DXA for fat in obesity is high [42,43], and errors in fat-free mass in the 0.7–1.9 kg range are typical [39,44], the same order as the change we report. That bioimpedance and DXA-derived muscle fractions run higher than computed tomography or magnetic resonance imaging estimates in randomized trials points the same way [11].

Two observations are consistent with, but do not demonstrate, the view that the early fat-free mass signal does not represent clinically important muscle loss. First, the height-normalized indices show the cohort moving towards a leaner phenotype: FFMI fell 1.22 kg/m^2^ and remained roughly 6.7 units above the thresholds used to screen for low muscle mass [31]. Second, in the matched analysis the FFM share of loss was largest in the first 4 weeks (about 45%) and smaller thereafter, a pattern compatible with the early glycogen and fluid shifts of negative energy balance; this remains an interpretation rather than a demonstrated mechanism, because the device cannot separate water from tissue and the later interval estimates were imprecise. Cohorts with DXA and dynamometry have shown the same shape as preserved or improved function: in a 106-patient semaglutide cohort, fat-free mass fell 3 kg to month 7 then stabilized, grip strength rose 4.5 kg, and the prevalence of sarcopenic obesity fell from 49% to 33% [17]; and in a 52-patient real-world tirzepatide cohort, fat-free mass and grip strength were unchanged [18]. Our study cannot make this argument directly, because muscle function was not measured, which is the principal gap between this cohort and the studies best positioned to answer the lean mass question. A vector-based bioimpedance analysis can separate fluid removal from fat loss, but it requires the resistance and reactance that a single-frequency, foot-to-foot device does not resolve; bioimpedance spectroscopy or a phase-sensitive device would be needed to apply it here [45].

The concern the field is trying to resolve is not fat-free mass in kilograms but muscle mass, quality, and function, and whether their loss is adaptive or maladaptive for a smaller body [15,16,46,47]. Under current diagnostic frameworks the diagnosis of sarcopenia rests on muscle strength rather than mass [48], and sarcopenic obesity is defined by adiposity combined with low muscle function and mass [49], which is why the absence of a functional measure is a central rather than a peripheral limitation here. That distinction matters most in older and more frail patients, in whom incretin-related lean loss has been compared in magnitude to more than a decade of ageing [50]. The mitigation strategies under active study, comprising adequate protein intake and resistance exercise [50,51,52], now being tested in a randomized trial of protein and resistance exercise during semaglutide and tirzepatide therapy [53], and the pharmacological approaches in preclinical development, including myostatin and activin blockade [54] and 15-PGDH inhibition [55], are all premised on lean tissue loss being at least partly preventable. Whether fat-free mass loss predicts weight regain is also unsettled: one cohort found a modest association [56] and another none once appetite changes were accounted for [57]. The composition of loss is shaped mostly in the first weeks and differences narrow thereafter [58], the very window this study covers.

The metabolic changes accompanying weight loss are consistent with the wider cardiometabolic profile of incretin therapy [19,59,60], and about three-quarters of the tirzepatide-associated fall in serum uric acid in SURMOUNT-1 was statistically mediated by weight loss [59]. Cardiovascular outcome data for tirzepatide in type-2 diabetes have now been reported [61]. The absence of detectable effect modification by baseline hepatic steatosis is reassuring at first sight, but this null should be read with its precision in mind (the 95% confidence interval for the between-group difference in fat mass change spanned −2.6 to 2.4 kg, so a clinically relevant difference cannot be excluded) and with the imprecision of the exposure classification in mind, because ultrasound grading is a coarse proxy for liver fat content [62,63]. In SURPASS-3 magnetic resonance imaging, tirzepatide reduced liver fat content by 8.09% against 3.38% for insulin degludec, with the reduction related to baseline liver fat and visceral fat [64,65], so a liver-fat-dependent difference in the adipose response is biologically plausible and would require quantitative imaging to detect.

Strengths of this study include its real-world setting, a cohort in which the composition question is addressed with several hundred rather than a few dozen patients, the focus on the early treatment period with granular time points at 4, 8, and 12 weeks, mixed-effects models that make full use of all available measurements rather than only complete cases, together with a sensitivity analysis for informative dropout, the integration of baseline hepatic phenotyping, and an explicit audit of what the measurement method can and cannot support.

### Limitations

Several limitations should be considered. First and most important, bioimpedance is convenient but is not the gold standard for body composition, and because it derives fat-free mass from body water it is sensitive to the fluid shifts that accompany rapid weight loss; dual-energy X-ray absorptiometry or magnetic resonance imaging were not available. Second, follow-up measurements were available for 268 of the 476 patients and for 111 at 12 weeks, so the estimates are available-data, on-treatment estimates rather than the trajectory of the full cohort, and patients entering the models were slightly heavier at baseline than those without follow-up. Completers and non-completers were similar on measured baseline characteristics and covariate adjustment did not change the estimate, but we could not test whether missingness was related to the unobserved weight change itself. Informative dropout, for example if patients losing less weight discontinued earlier, cannot be excluded; the delta-adjusted analysis indicates that the main finding is robust to substantial departures from the missing-at-random assumption, but the trajectory of patients who did not return is unknown. Third, the study was conducted at a single center without a comparator arm, and treatment was not randomized, so no comparative or causal claim can be made about tirzepatide relative to other agents; the changes described were observed during treatment and cannot all be attributed to tirzepatide. Fourth, the paired laboratory subset was modest in size. Fifth, the tirzepatide dose received at each visit, adherence, dietary intake (including protein), and physical activity were not available for analysis, and adverse effects were not analyzed; each can influence both the magnitude and the composition of weight loss. Finally, comorbidities were taken from recorded diagnoses and are likely to be under-ascertained, and ultrasound grading of steatosis was qualitative and performed by a single radiologist without assessment of reproducibility.

## 5. Conclusions

In routine care, early weight loss during tirzepatide treatment was predominantly fat mass, and the BIA-derived FFM fraction of about 30% was numerically close to the one-fourth commonly cited for negative energy balance; weight loss was accompanied by improvement in several metabolic parameters. Because the device-derived FFM is a fixed function of total body water, these data cannot distinguish water from lean tissue or skeletal muscle, and they describe patients who remained on treatment and returned for measurement. Controlled studies with reference body-composition methods and measures of muscle function are needed to establish the composition of early weight loss with tirzepatide.

## Figures and Tables

**Table 1:** Baseline characteristics of the tirzepatide cohort. Values are mean plus or minus standard deviation or number (percentage).

Variable	Mean ± SD or n (%)	n
n (tirzepatide monotherapy)	476	
Age, years	40.3 ± 10.8	473
Female sex	312 (65.5%)	474
Weight, kg	106.4 ± 19.2	476
Body-mass index, kg/m^2^	38.7 ± 5.7	476
Waist circumference, cm	117.8 ± 13.9	349
Fat mass, kg	45.0 ± 11.8	421
Body fat, %	42.0 ± 6.7	422
Fat-free mass, kg	61.7 ± 12.7	421
Total body water, kg	45.2 ± 9.2	422
Comorbidities		
Diabetes mellitus	54 (11.3%)	476
Insulin resistance	43 (9.0%)	476
Hypertension	14 (2.9%)	476
Coronary artery disease	7 (1.5%)	476
Hyperlipidemia	6 (1.3%)	476
Obstructive sleep apnea	6 (1.3%)	476
Baseline laboratory		
HbA1c, %	5.8 ± 1.0	385
Glucose, mg/dL	103.4 ± 29.5	396
Insulin, mIU/L	26.6 ± 22.4	368
HOMA-IR	6.8 ± 6.4	362
Total cholesterol, mg/dL	209.6 ± 44.3	393
LDL cholesterol, mg/dL	134.3 ± 31.9	391
HDL cholesterol, mg/dL	50.6 ± 11.4	390
Triglycerides, mg/dL	176.3 ± 121.8	392
ALT, U/L	31.6 ± 24.0	392
AST, U/L	25.0 ± 11.3	392
GGT, U/L	32.8 ± 23.0	364
Uric acid, mg/dL	6.1 ± 1.5	383
Creatinine, mg/dL	0.8 ± 0.2	391
eGFR, mL/min/1.73 m^2^	106.5 ± 16.5	386
TSH, mIU/L	2.5 ± 1.4	389
CRP, mg/L	9.8 ± 8.5	215
Ultrasonography		
Hepatic steatosis grade 0	103 (27.1%)	380
Hepatic steatosis grade 1	123 (32.4%)	380
Hepatic steatosis grade 2	114 (30.0%)	380
Hepatic steatosis grade 3	40 (10.5%)	380
Hepatic steatosis present	277 (72.9%)	380
Thyroid nodule	168 (43.9%)	383

Comorbidities are diagnoses recorded in the clinical record. HOMA-IR, homeostatic model assessment of insulin resistance; ALT, alanine aminotransferase; AST, aspartate aminotransferase; GGT, gamma-glutamyl transferase; eGFR, estimated glomerular filtration rate; TSH, thyroid-stimulating hormone; CRP, C-reactive protein.

**Table 2:** Body-composition trajectory over 12 weeks. Values are estimated marginal means from linear mixed-effects models. *p* refers to the overall effect of time (likelihood-ratio test).

Parameter	Baseline	Week 4	Week 8	Week 12	Change at 12 wk	*p*
Weight, kg	108.2	102.9	99.5	97.0	−11.22 (−10.4%)	<0.001
Body-mass index, kg/m^2^	39.2	37.3	36.1	35.2	−4.02 (−10.2%)	<0.001
Waist circumference, cm	119.7	115.5	111.2	110.4	−9.39 (−7.8%)	<0.001
Fat mass, kg	47.1	44.3	40.7	39.2	−7.90 (−16.8%)	<0.001
Body fat, %	42.7	41.9	40.0	39.3	−3.34 pts	<0.001
Fat-free mass, kg	62.6	60.3	60.4	59.2	−3.35 (−5.4%)	<0.001
Total body water, kg	45.8	44.1	44.2	43.3	−2.46 (−5.4%)	<0.001

Number contributing at baseline, week 4, week 8, and week 12: 268, 235, 128, and 111 for weight and 222, 189, 96, and 86 for fat mass. Change at 12 weeks is the estimated marginal difference from baseline; the value in parentheses is the percentage change, except for body fat, which is expressed in percentage points (pts). Estimates are available-data, on-treatment estimates (Figure 1).

**Table 3:** Proportion of patients reaching categorical weight loss thresholds at each visit (complete-case).

Visit	n	≥5%	≥10%	≥15%	Median % Change
Week 4	235	44.7%	3.4%	0.4%	−4.7%
Week 8	128	83.6%	26.6%	4.7%	−8.1%
Week 12	111	89.2%	57.7%	10.8%	−10.7%

Proportions are among patients with a weight measurement in the corresponding window. Because these are complete-case estimates, they are subject to the same attrition as noted for the primary analysis. A loss of at least 5% is associated with meaningful cardiometabolic improvement and is the threshold required for anti-obesity drug approval, whereas losses of at least 10% to 15% are associated with remission of steatohepatitis, obstructive sleep apnea and type-2 diabetes [35].

**Table 4:** Derived body-composition indices and the decomposition of weight loss.

Visit	FFMI, kg/m^2^	FMI, kg/m^2^	FM:FFM Ratio	TBW/FFM, %	Cumulative Fat Share, %	Interval Fat Share, %
Baseline	22.51	17.11	0.769	73.1		
Week 4	21.69	16.11	0.748	73.1	53.7	53.7
Week 8	21.69	14.83	0.690	73.1	74.4	106.1
Week 12	21.29	14.32	0.677	73.1	70.4	57.1

Indices were computed from each participant’s recorded height (cohort mean 1.66 m). FFMI, fat-free mass index; FMI, fat-mass index. Cumulative fat share is the fat mass change from baseline divided by the weight change from baseline; interval fat share is the same ratio computed between consecutive visits. The interval share exceeds 100% between weeks 4 and 8 because the modeled fat-free mass estimate rose slightly over that interval; this reflects the use of separately fitted models, and matched-subset estimates with 95% confidence intervals are given in Section 3.4 and Supplementary Table S4. TBW/FFM is constant at 73.1% with a fixed coefficient of approximately 0.731. The indices were computed from each participant’s individually recorded height and then averaged, so they differ slightly from ratios of the marginal means in Table 2; the cumulative and interval fat shares were computed from the unrounded model estimates rather than the rounded values shown.

**Table 5:** Laboratory values at baseline and at approximately 12 weeks in the paired subset. Values are mean plus or minus standard deviation.

Parameter	Baseline	~12 Weeks	n	*p* (*t*-Test)	*p* (Wilcoxon)
HbA1c, %	5.8 ± 1.0	5.3 ± 0.6	58	<0.001	<0.001
ALT, U/L	31.3 ± 23.0	26.6 ± 16.5	60	0.073	0.015
AST, U/L	24.8 ± 12.8	21.9 ± 8.2	61	0.059	0.064
GGT, U/L	31.4 ± 21.7	23.9 ± 19.4	58	0.020	<0.001
Total cholesterol, mg/dL	206.0 ± 43.9	189.3 ± 43.8	59	0.008	0.004
LDL cholesterol, mg/dL	128.4 ± 33.4	122.4 ± 31.3	59	0.152	0.106
HDL cholesterol, mg/dL	51.6 ± 12.6	50.4 ± 14.9	59	0.385	0.314
Triglycerides, mg/dL	178.1 ± 92.5	126.6 ± 48.6	57	<0.001	<0.001
Uric acid, mg/dL	6.2 ± 1.6	5.6 ± 1.4	58	<0.001	<0.001
Creatinine, mg/dL	0.8 ± 0.3	0.8 ± 0.3	61	0.810	0.754
eGFR, mL/min/1.73 m^2^	102.9 ± 20.0	103.4 ± 20.8	59	0.749	0.723

*p* (*t*-test), paired *t*-test; *p* (Wilcoxon), Wilcoxon signed-rank test. Paired differences departed from normality (Shapiro–Wilk *p* < 0.05) for HbA1c, ALT, AST, GGT, LDL cholesterol, triglycerides, uric acid, and eGFR.

**Table 6:** Effect modification by baseline hepatic steatosis: 12-week change by group and interaction with time.

Parameter	Steatosis Absent	Steatosis Present	n	*p* (Interaction)
Fat mass, kg	−7.99	−8.11	197	0.322
Fat-free mass, kg	−3.30	−3.12	197	0.263
Weight, kg	−10.79	−11.34	239	0.668
Body fat, %	−3.99	−3.31	198	0.412

Values are 12-week estimated marginal changes from baseline within each group. *p* is for the time-by-steatosis interaction (likelihood-ratio test). The interaction test could detect between-group differences of approximately 1.3–2.2 kg at 80% power. Between-group differences in 12-week change (present minus absent, 95% CI): fat mass −0.12 (−2.61 to 2.36) kg; fat-free mass 0.17 (−2.17 to 2.52) kg; weight −0.55 (−1.94 to 0.85) kg; body fat 0.68 (−1.35 to 2.72) percentage points.

## Data Availability

The data presented in this study are available on reasonable request from the corresponding author. The data are not publicly available due to privacy and institutional restrictions.

## Supplementary Materials

The following supporting information can be downloaded at: https://www.mdpi.com/article/10.3390/jcm15218377/s1, Table S1: The present cohort in the context of published body-composition studies of incretin therapy; Table S2: Visit-level availability of measurements; Table S3: Baseline characteristics by inclusion in the longitudinal models and by availability of a 12-week measurement; Table S4: Matched-subset decomposition of weight loss; Table S5: Delta-adjusted sensitivity analysis for informative dropout; Table S6: Weight change by baseline obesity class.

## Abbreviations

The following abbreviations are used in this manuscript:

BIA	Bioelectrical impedance analysis
BMI	Body-mass index
CT	Computed tomography
DXA	Dual-energy X-ray absorptiometry
EMM	Estimated marginal mean
FFM	Fat-free mass
FFMI	Fat-free mass index
FMI	Fat-mass index
GGT	Gamma-glutamyl transferase
GIP	Glucose-dependent insulinotropic polypeptide
GLP-1	Glucagon-like peptide-1
MAR	Missing at random
MRI	Magnetic resonance imaging
RECORD	REporting of studies Conducted using Observational Routinely-collected health Data
STROBE	Strengthening the Reporting of Observational Studies in Epidemiology
TBW	Total body water
