# Supplementary material for: Early Body-Composition Trajectory During Tirzepatide Treatment for Obesity: A Real-World Bioimpedance Cohort Study
Source: J Clin Med. 2026 Oct 28;15(21):8377. doi: 10.3390/jcm15218377 (PMC13764577; doi:10.3390/jcm15218377)
Supplement: Supplementary file 1 [file jcm-15-08377-s001.zip › jcm-4537122-supplementary.pdf]

Supplementary Materials

Early Body Composition Trajectory During Tirzepatide Treatment for Obesity: A Real-World Bioimpedance Cohort Study

Table S1. The present cohort in the context of published body-composition studies of incretin therapy.

Study	n	Agent	Follow-up	Method	Weight %	Fat mass %	FFM/lean %	Lean fraction of loss %	Muscle function
Present cohort	476 enrolled; 268 in weight model; 111 at week 12	Tirzepatide	12 weeks	Foot-to-foot BIA (TBF-300)	-10.4	-16.8	-5.4	31.7 (matched subset, n = 85)	Not measured
SURMOUNT-1 DXA substudy (Look 2025)	160	Tirzepatide vs placebo	72 weeks	DXA	-21.3	-33.9	-10.9	~25	Not measured
SEMALEAN (Alissou 2025)	106	Semaglutide 2.4 mg	12 months	DXA	-13	-18	-3 kg (then stable)	Not reported	Grip +4.5 kg
Real-world tirzepatide (Blanco Anesto 2026)	52	Tirzepatide (mean 5.1 mg/wk)	>=4 months	BIA	-9.7	-16.4	-2.3 (p=0.052)	Not reported	Grip unchanged
Incretin composition series (Bhatti 2025)	269	Semaglutide or tirzepatide	6 and 12 months	BIA (InBody 370S)	-10.7 (6 mo); -22.0 (12 mo)	Reduced (p<0.001)	Preserved with lifestyle	Not reported	Not measured
Systematic review (Batsis 2026)	35 RCTs	Liraglutide/semaglutide/tirzepatide/dulaglutide	Median 26 wk	BIA/DXA/CT/MRI	Varies	Varies	Varies	>25 (method-dependent)	Varies
Network meta-analysis (Karakasis 2024)	22 RCTs (2258)	GLP-1 RA and co-agonists	Varies	DXA or BIA	-3.55 kg	-2.95 kg	-0.86 kg	~25	Not measured

The studies differ in body composition method (BIA, DXA, CT or MRI), agent, dose, follow-up and outcome definition, and BIA-derived fat-free mass, DXA lean mass and muscle-related outcomes are not equivalent measures. The table is provided for context and is not intended as a quantitative comparison. BIA, bioelectrical impedance analysis; DXA, dual-energy X-ray absorptiometry; CT, computed tomography; MRI, magnetic resonance imaging; RCT, randomized controlled trial.

Table S2. Visit-level availability of measurements.

Variable	Valid values in full cohort (n = 476): baseline / week 4 / week 8 / week 12	Patients in longitudinal model	Model measurements: baseline / week 4 / week 8 / week 12
Body weight	476 / 235 / 128 / 111	268	268 / 235 / 128 / 111
Fat mass	421 / 217 / 115 / 103	222	222 / 189 / 96 / 86
Fat-free mass	421 / 217 / 115 / 104	222	222 / 189 / 96 / 87
Total body water	422 / 217 / 115 / 104	223	223 / 189 / 97 / 87
Body fat, %	422 / 217 / 115 / 104	223	223 / 189 / 97 / 87
Waist circumference	349 / 186 / 99 / 83	167	167 / 142 / 76 / 52

A patient entered a longitudinal model when a valid baseline value and at least one valid follow-up value within 126 days were available for that variable. For the composition variables, some patients had valid follow-up values but no valid baseline value and therefore did not enter the model. Of the 208 patients without a valid follow-up weight within 126 days, 15 were measured again only after this window.

Table S3. Baseline characteristics by inclusion in the longitudinal models and by availability of a 12-week measurement.

Characteristic	In weight model (n = 268)	Baseline only (n = 208)	p	12-week measurement (n = 111)	No 12-week measurement (n = 365)	p
Age, years	40.9 ± 10.8	39.4 ± 10.7	0.109	40.8 ± 10.2	40.1 ± 11.0	0.399
Female sex, n (%)	182 (67.9)	130 (63.1)	0.320	71 (64.0)	241 (66.4)	0.721
Weight, kg	108.2 ± 19.9	104.0 ± 18.1	0.027	108.3 ± 21.4	105.8 ± 18.5	0.377
Body mass index, kg/m2	39.2 ± 5.7	38.0 ± 5.7	0.009	38.9 ± 5.7	38.6 ± 5.7	0.529
Body fat, %	42.7 ± 6.8	41.1 ± 6.6	0.010	41.8 ± 7.3	42.0 ± 6.6	0.892
Fat mass, kg	47.1 ± 12.6	42.6 ± 10.4	<0.001	46.0 ± 12.9	44.7 ± 11.5	0.230
Fat-free mass, kg	62.4 ± 12.7	60.9 ± 12.6	0.145	63.6 ± 14.1	61.2 ± 12.2	0.229
Waist circumference, cm	119.6 ± 14.6	115.8 ± 12.9	0.021	119.3 ± 16.1	117.4 ± 13.3	0.475
Diabetes mellitus, n (%)	31 (11.6)	23 (11.1)	0.978	13 (11.7)	41 (11.2)	1.000
Insulin resistance, n (%)	29 (10.8)	14 (6.7)	0.167	11 (9.9)	32 (8.8)	0.858
Hypertension, n (%)	7 (2.6)	7 (3.4)	0.834	4 (3.6)	10 (2.7)	0.880
Hepatic steatosis present, n/N (%)	177/239 (74.1)	100/141 (70.9)	0.586	76/106 (71.7)	201/274 (73.4)	0.843

Values are mean ± standard deviation or n (%). p from the Mann-Whitney U test (continuous variables) or the chi-square test (categorical variables). Body composition variables were available in a subset of patients.

Table S4. Matched-subset decomposition of weight loss.

Interval	n	Mean change in weight, kg	Mean change in fat mass, kg	Mean change in FFM, kg	Fat share of loss, % (95% CI)	FFM share of loss, % (95% CI)	Individual FFM fraction, median (IQR), %
Baseline to week 4	187	-5.06	-2.78	-2.29	54.9 (41.9 to 66.9)	45.1 (32.4 to 58.1)	37.1 (9.7 to 68.5)
Baseline to week 8	92	-8.84	-7.14	-1.71	80.8 (67.2 to 95.9)	19.3 (4.3 to 32.9)	28.6 (0.1 to 49.1)
Baseline to week 12	85	-11.59	-7.91	-3.68	68.3 (56.1 to 80.8)	31.7 (19.5 to 44.1)	28.8 (10.8 to 51.5)
Week 4 to week 8	83	-3.57	-3.32	-0.25	93.2 (61.1 to 131.9)	7.1 (-32.7 to 37.5)	
Week 8 to week 12	62	-2.80	-1.94	-0.85	69.1 (17.8 to 117.0)	30.4 (-19.1 to 83.1)	

Analyses were restricted to records in which device weight equaled fat mass plus FFM within 0.5 kg (847 of 856 records). Shares are ratios of summed changes; 95% confidence intervals from 4000 bootstrap resamples of patients. The individual FFM fraction is reported among patients who lost weight. FFM is the bioimpedance-derived estimate. CI, confidence interval; FFM, fat-free mass; IQR, interquartile range.

Table S5. Delta-adjusted sensitivity analysis for informative dropout.

Assumed shortfall (δ), kg	Modeled population (n = 268; 157 imputed): mean change, kg (95% CI)	Mean change, %	Full cohort (n = 476; 365 imputed): mean change, kg (95% CI)	Mean change, %
0 (missing at random)	-11.22 (-12.09 to -10.35)	-10.5	-11.22 (-12.09 to -10.35)	-10.7
1	-10.64 (-11.53 to -9.74)	-9.9	-10.46 (-11.34 to -9.57)	-10.0
2	-10.05 (-10.95 to -9.12)	-9.3	-9.69 (-10.58 to -8.78)	-9.2
3	-9.46 (-10.36 to -8.50)	-8.8	-8.92 (-9.82 to -8.00)	-8.5
4	-8.88 (-9.75 to -7.89)	-8.2	-8.15 (-9.06 to -7.22)	-7.8
5	-8.29 (-9.19 to -7.27)	-7.7	-7.39 (-8.29 to -6.43)	-7.0

8	-6.54 (-7.53 to -5.49)	-6.0	-5.09 (-5.98 to -4.08)	-4.8

For patients without a 12-week weight, the value predicted by the linear mixed-effects model under the missing-at-random assumption (fixed effect plus the patient's predicted random intercept) was shifted by δ kg towards less weight loss. In the full cohort, patients without any follow-up weight were assigned the fixed-effect prediction plus δ. Percent change is the mean of individual percentage changes. 95% confidence intervals from 200 bootstrap resamples of patients.

Table S6. Weight change by baseline obesity class.

**Body mass index class**	**Patients in model**	**Measured at week 12**	**Baseline weight (EMM), kg**	**Change at 12 weeks, kg**	**Change at 12 weeks, %**
<35 kg/m2	61	26	90.7	-9.13	-10.3
35 to 39.9 kg/m2	104	45	102.8	-10.77	-10.3
≥40 kg/m2	103	40	123.9	-13.03	-10.2

Estimates from linear mixed-effects models with a time-by-class interaction. Interaction p = 0.004 for absolute weight change (kg) and p = 0.565 for percentage weight change. EMM, estimated marginal mean.
